# A Theory Planned Behaviour of Study on Improving Abdominal Bloating among the Malays Population: A Qualitative Study

**DOI:** 10.21315/mjms2022.29.6.8

**Published:** 2022-12-22

**Authors:** Nurzulaikha Abdullah, Yee Cheng Kueh, Garry Kuan, Fatan Hamamah Yahaya, Yeong Yeh Lee

**Affiliations:** 1 Biostatistics and Research Methodology Unit, School of Medical Sciences, Universiti Sains Malaysia, Kelantan, Malaysia; 2 Exercise and Sports Science, School of Health Sciences, Universiti Sains Malaysia, Kelantan, Malaysia; 3 Department of Life Sciences, Brunel University, London, United Kingdom; 4 School of Distance Education, Universiti Sains Malaysia, Pulau Pinang, Malaysia; 5 Department of Medicine, School of Medical Sciences, Universiti Sains Malaysia, Kelantan, Malaysia; 6 GI and Motility Unit, Hospital Universiti Sains Malaysia, Kelantan, Malaysia

**Keywords:** theory of planned behaviour, health belief, health promoting behaviour, abdominal bloating

## Abstract

**Background:**

Abdominal bloating (AB) is a common medical complaint known to diminish patients’ quality of life. However, lifestyle and behavioural changes could mitigate its expression and severity. This study sought to explore the health beliefs, intentions and health-promoting behaviours among people with AB in Kelantan, Malaysia.

**Methods:**

The theory of planned behaviour (TPB) was employed to explore the factors that influence the management of adults with AB, namely health beliefs and intentions. An interview guide was developed by adapting the TPB and the findings of prior studies concerning the factors associated with AB management. All eligible participants took part in in-depth interviews.

**Results:**

The mean age of the participants was 32.5 years old (standard deviation [SD] = 14.19 years old) and the majority of participants were female (58.3%). The themes and sub-themes that emerged following the application of the TPB framework represented the qualitative results of this study, which indicated that the health beliefs, intentions and health-promoting behaviours observed among people with AB were closely related.

**Conclusion:**

The findings of this study suggest that the adoption of a healthy lifestyle could be an effective means of improving AB. Thus, it is vital to implement a health education programme that promotes health-related beliefs and intentions in order to trigger health-promoting behaviour among people with AB.

## Introduction

Abdominal bloating (AB) is a common and disturbing medical complaint with only limited treatment options, which means that further investigations are required into its causes and potential treatments (1). Interestingly, AB can occur in healthy individuals as well as those with known medical conditions (2). AB and distension have been defined as a ‘subjective sensation of abdominal swelling’ and an ‘actual increase of abdominal girth’, respectively (3). In Asian communities, people have traditionally reported experiencing both symptoms (i.e. AB and distention) to feel like having ‘a lot of air in the stomach’, as suggested by Seo et al. (4). Moreover, although AB and distention have distinctive patterns, they also share a lot of similarities, which renders them difficult to distinguish for most people.

AB has been hypothesised to occur due to mechanisms potentially associated with functional gastrointestinal (GI) disorders such as mucosal immune activation, generation of excessive intestinal gas, food sensitivity, constipation and other possible triggers, including psychological factors, obesity, medications and other medical conditions (2, 5, 6). Thus, as the condition varies from one individual to another, a proper diagnosis is required to identify an appropriate treatment so that the AB can be resolved quickly and efficiently.

The Rome IV diagnostic criteria for functional AB include: i) recurrent AB or visible distension for at least one day per week; ii) the onset of symptoms at least 6 months prior to diagnosis; iii) the presence of symptoms for at least 3 months and iv) insufficient criteria for a diagnosis of irritable bowel syndrome (IBS), functional dyspepsia or any other functional GI disorder that may coexist with mild abdominal pain and minor bowel disorders (7). In addition, AB and distension are both recognised as common symptoms of IBS according to the Rome IV diagnostic criteria (8).

A study conducted in the United States found that at least 65% of patients with AB exhibited moderate to severe symptoms, while up to 54% complained of decreased physical activity and 43% reported taking or requiring medication due to AB (4, 9). Studies conducted in the United States and Canada have identified the prevalence of AB to range from 66% to 90%, while in Asian countries its prevalence has been found to vary between 15% and 23% (8, 9). While it is known to be a common complaint, AB is considered difficult to manage effectively due to different people weighting its severity and consequences differently (6). Moreover, AB can be an underlying marker of chronic diseases such as gastritis and stomach cancer (10). Although there is no universally recognised treatment for AB, several interventions have been developed over the last 5 years (11). However, as AB continues to adversely affect the quality of life of numerous individuals worldwide, it is a concern that many clinicians and healthcare providers wish to better address.

The determinants of health-related behaviour often simultaneously prompt changes in such behaviour (12, 13). Due to the multifactorial influences on the outcomes of behavioural changes, various psychological theories have been developed to predict health-related behaviours, including the health belief model (HBM) (12), theory of planned behaviour (TPB) (13), theory of reasoned action (TRA) (13) and Pender’s health promotion (PHP) model (14). As a practical example, in patients with type 2 diabetes, the ability to self-manage their disease has been reported to have a profound positive influence on their quality of life (15, 16). Similar to the case of diabetes, AB can be influenced by factors that might differ from the clinical perspective, such as the patient’s perception and culture (7). For instance, the food choices (sweet and spicy) and socio-demographic factors of Kelantanese Malay people vary when compared with those of people from other areas. In addition, the perception that AB is common and does not require medication can also influence its management.

In the present study, the TPB has been adapted to reveal the beliefs on behalf of patients that may trigger action to help improve the symptoms of AB. The study was motivated by the belief that more research is required to understand patients’ health beliefs, intentions and health-promoting behaviours when it comes to dealing with AB and reducing its impact on their quality of life. Thus, this study sought to elucidate patients’ responses and beliefs based on their experiences concerning the management of AB and the associated treatment intentions.

## Methods

### Research Design

A qualitative research design involving semi-structured interviews was employed to explore the participants’ perceptions of AB based on their life experiences. Moreover, this study adopted the qualitative phenomenological framework developed by Creely (17), which aligned with the aim of understanding given phenomena (perception, intention and management of AB) on the basis of lived experiences.

### Study Population and Eligibility

After providing informed consent to participate, all patients who presented with a complaint of AB at the Gastroenterology Clinic of Hospital Universiti Sains Malaysia (HUSM), Kota Bharu, Kelantan were screened for eligibility for the present study. The inclusion criteria for the study were as follows: aged 18 years old or above, no prior abdominal surgery, not on drugs that cause or worsen AB (e.g. opiates) and no history of any psychiatric illness (e.g. schizophrenia).

### Qualitative Study Framework

The theoretical framework chosen for this study was the TPB (13). The psychosocial behavioural model of the TPB represents an extension of the TRA (13). In addition to the original domains of the TRA (attitude, attention and behavioural relationships), the TPB includes the indirect measurement of behavioural beliefs, normative beliefs and perceived behavioural control. Behavioural intention is a key determinant of the TPB, especially its influence on disease outcomes such as quality of life (18). Francis et al. (19) suggested that attitude, subjective norms and perceived behavioural control, which are the domains representing health-related beliefs, can increase an individual’s likelihood of taking action. As the TPB proposes that behaviour is intentional, its three core domains all correspond to intention (13), which indicates that the TPB is suitable for use with regard to behavioural predictions.

The present study focused on predicting the health-promoting behaviours of individuals with AB on the basis of three aspects of health-related beliefs, namely attitudes, subjective norms and perceived behavioural controls (19). First, the attitudes in question included the following: global evaluations, positive or negative evaluations, evaluative judgements concerning advantages and disadvantages, and whether the individual was in favour of doing the behaviour in question. Second, the subjective norms referred to perceived social pressures, perceived wishes of important others and perceptions of social pressures related to AB. Third, the perceived behavioural controls included an individual’s perceived confidence and capacity as well as an individual’s belief in the control of the action in question. Furthermore, aside from the three above-mentioned aspects, the ‘intention’ domain (i.e. the goal or objective of self-management among the study participants) was also assessed.

### In-depth Interview

The in-depth interview method was chosen as the qualitative research method for this study. The content of the interviews was initially based on a topic guide developed on the basis of a literature review and the opinions of seven experts in the GI, psychometric and questionnaire development fields. The guide was then simplified into a series of open-ended questions. The first few interviews served as a pilot study to finalise the guide and practice. The focus of the interviews included the following: i) how the participants perceived AB; ii) how others viewed when they experienced AB; iii) how they controlled their recurrent symptoms; iv) their intentions to improve their AB and v) the management of their AB.

The participants were initially approached via telephone calls. They were asked to meet the investigator (the first author) at the clinic, where there was a quiet room dedicated to conducting the interviews. All of the interviews were conducted in the Malay language and audio-recorded with the participants’ consent. The interviews lasted approximately 40 min–60 min. Afterwards, all of the interviews were manually transcribed from the audio recordings and additional notes taken during the interviews. Further information was obtained from the participants via telephone calls when there were queries concerning the transcripts. In such cases, the audio recordings were also checked. For quality control purposes, the transcripts were independently reviewed by another researcher to ensure their accuracy.

The initial content analysis was performed using a quantitative approach to capture the occurrences (frequencies and percentages) of terms or phrases that were relevant to the concepts pre-determined to be associated with TPB. Next, the common concepts were determined based on the occurrences of the relevant statements. Subsequently, the interview transcripts were coded based on the pre-determined concepts, which were then categorised using aggregated quotes. Microsoft Word (Microsoft Corp., Redmond, WA, USA) was used to arrange the encoded data, while the descriptive data analysis was performed using Microsoft Excel (Microsoft Corp., Redmond, WA, USA).

When using the interview method, there is no need for a fixed sample size as long as data saturation is reached (20). In the present study, saturation was considered to be reached when there were recurring themes in the responses as well as rich, reliable and replicable qualitative results, which is in accordance with the findings of the literature review and the expert information previously gathered (21, 22). It was decided that at least 12 interviews would be conducted to ensure that trustworthy and rich information was obtained from the participants. The number of interviews were increased to 15 for testing and backup when further information might be required.

### Data Analysis

The analysis of the interview transcripts was performed using an inductive approach that allowed the researcher to identify patterns within the transcripts using thematic codes, which is known as a classical content analysis (23). The data were manually transcribed verbatim from the audio recordings to Microsoft Word documents. These interview transcripts and additional files such as participant profiles and field notes were organised properly to facilitate the data analysis. After the data were transcribed, they were coded and analysed. The coding process involved ‘deriving and developing concepts from data’, as suggested by Corbin and Strauss (24). These processes were performed immediately after each in-depth interview. The transcription process helped the researcher to gain a better understanding of the subject based on the repeated act of listening to and reading the interview data, while also allowing the researcher to become more familiar with the data (25).

The data coding process began once all of the data were transcribed. Ritchie et al. (25) defined the codes applied as keywords that are used to categorise or organise text, which is considered one of the essential aspects of qualitative research. Next, the codes were interpreted and grouped into categories, which were later mapped to identify the patterns and associations. The data were then further analysed and organised into themes and subthemes. The generated themes and subthemes were considered the results of this study.

To ensure the validity of the qualitative results, it was vital to make certain that the rigor and trustworthiness of those results aligned with the alternative terminology of truth value, consistency, neutrality and applicability. The goal was to accurately represent the participants’ experiences so as to ensure the credibility of the present study (26, 27). This included acknowledging the biases within the sampling, keeping meticulous records, engaging with other researchers to reduce any research biases and performing triangulation. Moreover, the rigor of the results was enhanced by obtaining feedback from the participants. Response validation was performed by reviewing and confirming the responses given by the participants to the interview questions and summarised at the end of each interview session. The applicability of the results to other settings was also considered. Moreover, the advice that was sometimes sought from qualitative experts helped to ensure the validity of the results. In sum, all four characteristics were considered throughout the study in order to increase the credibility of the obtained results.

### Ethical Approval

The present study was approved by the Human Ethics Committee of Universiti Sains Malaysia. In addition, all study procedures complied with the guidelines set out in the Declaration of Helsinki. A research information sheet was provided to all of the participants during the data collection phase to ensure that they fully understood the study. This research information sheet included the study’s purpose, procedures and potential risks and benefits. The participants were also informed that their participation was entirely voluntary and that they were free to withdraw from the study at any time without penalty. Finally, written informed consent was obtained from all of the participants. Some monetary incentives were provided to the participants to cover their transport costs and/or time.

## Results

As shown in Table 1, the ages of the participants ranged from 18 years old–58 years old (mean = 32.5 years old) and the majority (58.3%) of the participants were female. The participants all reported their AB to be bothersome.

The following five themes were identified in the interview data: i) attitudes towards the management of AB; ii) normative beliefs concerning AB; iii) perceived behavioural control of AB; iv) intentions concerning the management of AB and v) health-promoting behaviours. The participants’ verbatim quotations were analysed in the Malay language to increase the objectivity of the analysis. However, these quotations underwent forward and backward translation into the English language during the writing of this manuscript.

### Theme 1: Attitudes towards the Management of AB

One participant reported that the discomfort associated with having AB was not a good experience. Moreover, the experience was made worse by the additional symptoms brought on by the AB:

‘the feeling of not being comfortable, stress… it’s good to heal it… when bloated, it results in a headache, pain in abdominal area, so I want to find a way to cope with it.’

Some of the participants shared their opinions on how the symptoms of AB could be lessened through good lifestyle management, which they felt could help to prevent or relieve AB:

‘I agree with the opinion that when we care about our diet and lifestyle, it can help us to control the AB.’

### Theme 2: Normative Beliefs Concerning AB

Some of the participants revealed different opinions concerning the strategies available for relieving AB. They also reported social pressure and perceptions or wishes related to AB to be problematic. In addition, a number of the participants considered that AB was best treated in accordance with different people’s advice. The following statements illustration these differing beliefs:

‘It is really a dietary issue, so I asked my family and they told me to eat food that does not cause AB.’

‘People around us suggested putting on ointment to force the gas out, but when that actually happened, it made the people around us feel uncomfortable…’

‘...basically, I’m looking for advice from them, but it seems like my parents always advise me to eat on time, despite how busy I am.’

### Theme 3: Perceived Behavioural Control of AB

The participants also shared their perceived behavioural control over the improvement of their AB. Some were of the opinion that individuals should have confidence in their ability to perform the required behaviour, while others questioned whether every person would feel in control of the necessary action. Several participants suggested that AB is sometimes uncontrollable, whereas it can be controlled with better management at other times. For example, two participants reflected on how they were able to control the symptoms of their AB:

‘Controllable, it can still be controlled…’

‘It can still be controlled. I usually know the aetiology, it’s caused by myself, so I’m the one who can control it.’

By contrast, two participants who also suffered from other diseases (e.g. IBS) indicated that, while they had attempted to control the symptoms of their AB, their attempts had proved ineffective, which was part of the reason why having AB was so difficult:

‘It is uncontrollable. In fact, when I feel bloated, it’s hard for me, and it’s also hard to come to the hospital for regular treatment.’

‘It’s hard for me, as I need to go to the hospital frequently for my routine check-up.’

A number of other participants explained that they found it difficult to decide whether or not their condition was controllable because the severity of their AB symptoms differed from episode to episode.

### Theme 4: Intentions Concerning the Management of AB

The interviews included references to the participants’ intentions to improve their AB symptoms. In fact, all of the participants agreed that they intended to treat or improve their symptoms by better management of their daily routine, even when the severity of their symptoms is minimal:

‘I feel negative about it, (AB) is something that is not good for me... if something is negative, I do not want it to be in my life.’

‘It would be a good thing if I could recover from it.’

‘Yes, if I can, I want to recover from it, I need to prevent it. If I know the way to do so, then I have to work through it. But sometimes it just happens and there is nothing I can do to treat it.’

### Theme 5: Health-Promoting Behaviours

The participants described five health-promoting behaviours, namely: i) being physically active; ii) eating responsibly; iii) managing stress; iv) undergoing regular medical check-ups and v) engaging in self-treatment.

#### i) Being Physically Active

Some of the participants considered that being physically active could help to alleviate the symptoms of AB by improving digestion:

‘…I actively participate in sports and I eat at night. I do not know what contributes to no AB… but when I exercise, it helps to smooth the digestive process at night.’

In addition, a number of participants reported that not engaging in active exercise could make the symptoms of AB worse:

‘…I need to be active physically, to do exercise; however, I do not have enough time to do that… and so I feel bloated more frequently…’

#### ii) Eating Responsibly

From the participants’ perspective, eating responsibly involves eating normal-sized meals on a regular schedule and avoiding certain foods (e.g. instant foods):

‘I can reduce my AB episodes by eating on time, but if I eat more than on a regular basis or eat to a different schedule or eat instant food, then I will experience AB again and again…’

‘I prefer to eat more at one time, and during the long interval before the second meal, I do that at home, but at university, it’s common for me to miss my meal.’

Over time, some of the participants have tried certain diets thought to relieve the symptoms of AB, although the diets have often not proved helpful due to certain constraints, meaning that the participants had to rely on immediate relief medications (e.g. antacids):

‘I have my own diet plan, although it depends on the conditions I am in, as my working hours are not flexible. If I do not have enough time to take lunch, I will at least try to eat some biscuits. I also prepare some ENO (an antacid), so that once I feel bloated, I can take the ENO and not be disturbed fully.’

#### iii) Managing Stress

Several participants reported experiencing frequent stress to be a source of AB:

‘Perhaps it could affect it, too. Either stress or not enough sleep may have an adverse effect, as I slept less than two or three hours a day on average.’

‘Yeah... some people said that stress is the centre of all the problems.’

In addition, a number of participants revealed that experiencing AB can lead to stress and depression:

‘Erm… if I bloat, I do not like that feeling. I’m going to feel uncomfortable and it makes me depressed.’

‘Erm… my pain threshold is pretty low. So, even if I’ve had a little pain, I feel like it’s going to be worse, I’m going to feel emotional… I’m going to feel angry at even the slightest joke.’

‘It makes me suffer. I feel confused, stressed, for a short time, because I’ve had other things to take care of and not enough time.’

#### iv) Undergoing Regular Medical Check-Ups

The participants expressed how it is difficult to avoid frequent AB episodes if the condition has already reached a severe stage. As a consequence, they considered it important to be aware of their health status and to seek help from their healthcare provider when necessary:

‘I’m going to see a doctor. I am aware of the importance of this…’

‘I need to visit the hospital on a regular basis, so it is not good for me. Sometimes I have to go up to six times a month. It’s already my second or third time this month. I’m going to have trouble with my job soon.’

#### v) Engaging in Self-Treatment

Some participants discussed how self-treatment can help to deal with low to mild severity AB symptoms:

‘It is common and it can happen to anyone. I put some ointment on it and just wait for it to be relieved. Family help by preparing hot milk too…’

‘No one ever will help me, so the only thing I can do is self-massage.’

‘…Nothing can treat it instantly, if I use ENO… maybe it only works after two to three hours.’

Due to such experiences, most of the participants believed that self-treatment could help them to deal with minor AB symptoms, while support from the people around them could help to make the problems associated with AB slightly easier to manage.

Table 2 summarises the participants’ responses based on the adapted version of the TPB used in this study.

## Discussion

The present study sought to elucidate the beliefs, perceptions and intentions of the participants when it comes to lifestyle management as an alternative treatment for AB. The TPB was applied to understand the perceptions that might influence the self-management of AB symptoms by exploring the psychosocial factors of relevance to adult AB patients in Malaysia. Psychosocial factors are recognised as important in terms of understanding the pathophysiology and formulation of effective treatments for symptoms or diseases (28, 29). Some people with AB are unaware that a healthy lifestyle is important in relation to dealing with both AB symptoms and other lifestyle-related diseases, which results in them continuing to suffer from the associated symptoms. Thus, it is important to explore the potential of lifestyle management as a possible alternative treatment for AB.

Several studies concerning IBS have shown that the prevalence of AB is worrisome and, further, that it is more prevalent among females and obese people (4, 30). The association between women and AB can be logically explained by the hormonal effects of the menstrual cycle (4). In addition, eating habits and obesity are among the factors considered to potentially contribute to the increasing number of AB cases worldwide (4, 5) due to the linkage with the ‘gut’. Abdullah et al. (31) found that AB is commonly reported to be bothersome and to result in a reduced quality of life due to an impaired health status, mood disturbance, limitation of daily activities and increased need for healthcare. Therefore, the improvement of symptoms or diseases through lifestyle changes may help to increase the wellbeing of people who experience AB.

Irrespective of gender and underlying medical conditions, AB can cause significant distress to patients. For instance, among AB patients who did not have IBS, the majority reported their symptoms to be moderate to severe and, further stated that they had reduced their daily activities to some degree due to AB (31, 32). Moreover, AB has been found to be a significant predictor of the severity of IBS (30). Given that it has been reported to be the third most important reason for seeking medical care, prior studies have examined different aspects of interventions designed to treat the symptoms of AB in an effort to find a cure for it (33–36). The findings of the present study suggested lifestyle management to be a possible alternative treatment for AB.

In a meta-analysis, the TPB constructs were found to be used successfully to identify the psychosocial determinants of children’s physical activity and, further, to have explained 45% and 27% of the variance in children’s intentions and behaviours, respectively (37). The version of the TPB implemented in the study was based on a manipulation of the three core aspects, namely attitude (in favour of behaviour), subjective norm (social pressure to act in a certain way) and perceived behavioural control (power to control the action to be taken) (19). There has been an increase in reports concerning the implementation of the TPB in medical research since it was reported to help to more successfully explore the area of interest (13, 15, 18, 38). Furthermore, the TPB can help to improve compliance when used to develop strategies for helping clinicians to rule on the basis of guidelines and ensuring that people maintain healthy behaviours (19). Thus, the determination of the health beliefs (attitude, normative belief and perceived behavioural control) among people with AB could indicate alternative ways to promote the management of symptoms through suitable health-promoting behaviour.

Most cases of AB are modifiable or preventable through early identification and the modification of lifestyle choices. Positive beliefs and good intentions can motivate an individual to act in a certain way (13). Therefore, a healthy lifestyle could be facilitated through a combination of efforts to increase awareness, change behaviour and create an environment that promotes good health-related practices (39). Health-promoting behaviour is linked to a variety of variables that can enhance a person’s overall belief, thereby leading to behavioural changes, with the TPB being a useful concept in this regard.

The present study was limited by the fact that all of the participants were recruited from one major hospital in Malaysia. Thus, the results may not be reflective of the entire adult population of the country. However, the qualitative approach applied in this study provided new insights and understandings concerning health-related beliefs and intentions that may be associated with the self-management of AB from a local community perspective.

## Conclusion

The treatment of health-related issues such as obesity and AB is known to be linked to lifestyle management. This qualitative study found that people with AB exhibit different health-related beliefs (attitude, normative belief and perceived behavioural control) and intentions in terms of seeking help, preferably through health-promoting behaviour such as lifestyle manipulation, stress management and self-treatment, which enhance motivation and inspire action towards better health management. Thus, it is important for people to know more about AB and how to take action by adopting a healthier lifestyle, in addition to seeking medical and health treatment. Interventions that allow health practitioners to utilise new technologies (e.g. virtual hypnosis and imagery) in the treatment of AB and other diseases will likely prove fruitful.

## Figures and Tables

**Table 1:** The participants included in the study

Participant’s ID	Age (years old)	Gender	Marital status	Occupation	Comorbidities
1	45	Male	Married	Admin staff	Yes
2	42	Female	Married	University staff	Yes
3	21	Female	Single	University student	Yes
4	21	Female	Single	University student	Yes
5	52	Male	Married	Teacher	Yes
6	25	Female	Single	Job seeker	No
7	19	Female	Single	University student	No
8	20	Female	Single	University student	No
9	40	Female	Married	Admin officer	Yes
10	58	Male	Married	Farmer	Yes
11	29	Male	Single	Officer	No
12	18	Male	Single	Vocational student	No

**Table 2:** Emerging themes and subthemes adapting TPB

		Frequency	Participants response/sub-themes
Health belief	Attitude	10/12	AB symptom can be related to lifestyle
		12/12	AB is manageable through proper health management
	Subjective norm	11/12	People around them think that it is better to heal AB
	Perceived Behavioural control	10/12	Have complete control over AB with better management of health
Intention		12/12	There is a need to treat it
Health promoting behaviour		12/12	AB is manageable by: Physically active Diet management Stress management Health awareness Self-treatment
